# Molecular detection and genetic characterization of *Anaplasma marginale* and *Anaplasma platys*-like (*Rickettsiales: Anaplasmataceae*) in water buffalo from eight provinces of Thailand

**DOI:** 10.1186/s12917-020-02585-z

**Published:** 2020-10-08

**Authors:** Anh H. L. Nguyen, Sonthaya Tiawsirisup, Morakot Kaewthamasorn

**Affiliations:** 1 The international graduate course of Veterinary Science and Technology (VST), Faculty of Veterinary Science, Chulalongkorn University, Bangkok, 10330 Thailand; 2 Veterinary Parasitology Research Group, Faculty of Veterinary Science, Chulalongkorn University, Bangkok, 10330 Thailand; 3 Animal Vector-Borne Disease Research Unit, The Veterinary Parasitology Unit, Department of Veterinary Pathology, Faculty of Veterinary Science, Chulalongkorn University, Bangkok, 10330 Thailand

**Keywords:** *Anaplasma marginale*, *Anaplasma platys*, Detection, Genetic characterization, Thailand, Water buffalo

## Abstract

**Background:**

Anaplasmosis, an animal disease caused by rickettsial bacteria in the genus *Anaplasma*, is of considerable economic importance in livestock animals in many countries worldwide. The objectives of this study were to determine the identity, prevalence, and geographic distribution of *Ehrlichia* and *Anaplasma* in naturally infected water buffalo in Thailand using PCR amplification and sequencing of the 16S ribosomal RNA and heat shock protein groEL genes. A total of 456 buffalo blood samples from Thailand were investigated. Species identification and genetic differentiation of intra-population and inter-population with the global isolates were conducted based on nucleotide sequences. Interplay between the infection and host factors was also assessed.

**Results:**

Overall, 41% of water buffalo were found to be infected with rickettsial organisms in the family *Anaplasmataceae*, but *Ehrlichia* spp.*, Neorickettsia* spp., and *Wolbachia* spp. were not found in any of the sequenced samples in this study. Female buffalo were more frequently infected with bacteria in the family *Anaplasmataceae* than males [71 out of 176 females (40.3%) versus 11 out of 47 males (23.4%)]. The Odds Ratio value indicated that the risk of infection for female buffalo was 2.2-fold higher than that for males (*p* < 0.05). We detected three haplotypes of *A. marginale* 16S rRNA gene and they were placed in a clade that was closely related to the *A. marginale* in buffalo in China; and cattle in Thailand, Uganda, and China. Homology searching of groEL sequences against the GenBank™ database using the BLASTn algorithm revealed that the obtained sequences had a high percentage similarity (98.36–99.62%) to *A. platys* sequences. The groEL sequences of three *A. platys*-like isolates were clustered in the same clade as the *A. platys* from the tick *Rhipicephalus microplus* in China*.*

**Conclusions:**

Our data showed that the apparently healthy buffalo were naturally infected by bacteria in the family *Anaplasmataceae* at a relatively high prevalence. We also report the finding of *A. platys*-like infections in water buffalo in Thailand for the first time. Water buffalo serving as the reservoir host of anaplasmosis is of concern for managing the disease control and prevention in ruminants.

## Background

Water buffalo (*Bubalus bubalis*) is a multipurpose ruminant that contributes to livestock agriculture in Thailand, including farm operations, income insurance, capital formation, and food production [1]. Since buffalo in the rural areas of Thailand are frequently raised together with beef cattle, they might be exposed to the same vectors and environmental conditions to acquire transmissible bovine diseases. Nevertheless, compared to cattle, buffalo seldom show clinical symptoms, presumably owing to their breed resistance [2]. Thus, their potential to become asymptomatic reservoir hosts for tick-borne diseases has probably been underestimated.

Anaplasmosis is one of the most common tick-transmitted diseases in bovines worldwide. It is caused by obligatory intra-erythrocytic rickettsial organisms in the genus *Anaplasma* spp. [3]. In addition to *A. marginale*, *A. centrale* and *A. bovis* are also known to cause disease in cattle, while *A. phagocytophilum* (formerly *Ehrlichia phagocytophilum*) is a causative agent of human and animal granulocytic anaplasmosis and has been described from a broad range of animals, including goats, sheep, yaks, horses, dogs, cats, rodents, wild boars, foxes, birds, and reptiles [4]. *Anaplasma platys* (formerly *Ehrlichia platys*) is the etiological agent for infectious canine cyclic thrombocytopenia, and has been reported to also infect cattle and humans [5–8]. The principal clinical signs in *A. marginale*-infected cattle of over two-year-old include progressive anemia, fever, jaundice, loss of appetite, decreased milk production, abortion, and death [9]. Water buffalo show milder symptoms upon infection than cattle, with symptoms of progressive weakness, anorexia, high fever, tachycardia, labored respiration, and a pale mucus membrane [10]. In Thailand, there has been only one report of anaplasmosis in water buffalo, which was in the northeast provinces [11]. Anaplasmosis is not only biologically transmitted by ticks, but it is also mechanically transmitted by biting flies or blood-contaminated equipment. Additionally, transplacental transmission from an infected mother to her offspring has also been reported [12].

In Thailand, a series of surveys on bovine anaplasmosis using both conventional [13] and molecular methods has been conducted in beef and dairy cattle [14], and in ticks [15, 16] from different parts of Thailand. However, those studies were mainly focused on dairy cattle. In contrast, the prevalence, geographic distribution, and genetic diversity of anaplasmosis in water buffalo remain largely unknown and understudied. The present study, therefore, aimed to determine the prevalence, geographic distribution, and genetic characterization of tick-borne rickettsial organisms in water buffalo.

## Results

### Prevalence and distribution of tick-borne rickettsial organisms in buffalo in Thailand

In the present study, the prevalence of rickettsial organisms in the family *Anaplasmataceae* in buffalo from eight provinces in Thailand varied from 6 to 67.6% with an overall average of 41%. *Ehrlichia* spp., *Neorickettsia* spp. and *Wolbachia* spp. were not detected in any of the sequenced samples in this study. We detected anaplasmosis in water buffalo across different sampling sites and in every month that we conducted the sampling. Buffalo in Phatthalung had the highest prevalence of rickettsial organisms with more than two-thirds of the animals being positive. *Anaplasma platys*-like was detected in three out of 456 buffalo blood samples (0.66%), but was restricted to the Northern province of Lampang only (Table 1).

**Table 1:** Prevalence and geographic distribution of tick-borne rickettsial organisms in the family *Anaplasmataceae* in water buffalo from the eight sampled provinces in Thailand

Sampling site	Sampling date	No. tested	*Anaplasmataceae* positive (%)
Dong Luang, Mukdahan	Jan 2015	88	38 (43.2)
Mueng, Mukdahan	Jan 2016	61	40 (65.6)
Khamcha-i, Mukdahan	Dec 2017	36	7 (19.4)
Uthai Thani	May 2015	8	4 (50)
Lampang	June 2016	60	28 (46.7)^a^
Amnat Charoen	Dec 2016	21	5 (23.8)
Nong Bua Lamphu	Dec 2016	50	3 (6)
Phatthalung	April 2017	37	25 (67.6)
Surin	March 2018	13	2 (15.4)
Chachoengsao	June 2018	82	35 (42.7)
Overall		456	187 (41)

^a^Comprised of three isolates of *A. platys* out of 28 *Anaplasmataceae*-positive samples

### Association between infection with rickettsial organisms and the buffalo’s age and gender

Evaluation of the host factors associated with infection with rickettsial organisms (Table 2) revealed that the two age groups of buffalo (≤ 2 and >  2-y-old) had an equivalent prevalence (36.8%) of rickettsial organisms infection (32 positive out of 87 buffalo aged ≤2 y-old and 50 positive out of 136 buffalo aged > 2 y-old), indicating that pathogen infection proportions were age independent [Odds Ratio (OR) = 0.999] with no significant difference between them (*p* > 0.05). However, female buffalo were more frequently infected with bacteria in the family *Anaplasmataceae* than males [71 out of 176 females (40.3%) versus 11 out of 47 males (23.4%)]. The OR value also indicated that the risk of infection for female buffalo was 2.213-fold higher than that for males, and this was statistically significant (*p* < 0.05). This result is in contradiction to the previous findings in Pakistan, where male buffalo had a higher disease prevalence (26.25%) than females (16.62%), although this was from a larger sample size of 118 males and 617 females [17]. When the univariate general linear model was used to recheck the association between infection with rickettsial organisms and the gender or age of water buffalo, the univariate regression analysis indicated that there was an association between the buffalo’s gender and infection with rickettsial organisms (*p* = 0.032).

**Table 2:** Host factors associated with infection with rickettsial organisms in the family *Anaplasmataceae* in Thai water buffalo

Factor		***Anaplasmataceae***		OR	95% CI	***p***-value
		Positive	Negative			
**Age (y)**	≤ 2	32	55	0.999	0.572–1.746	0.998
	> 2	50	86			
**Gender**	Male	11	36	2.213	1.057–4.635	0.041*
	Female	71	105			

Asterisk indicates statistically significant

### Genetic relationship and phylogenetic analysis of *A. marginale* and *A. platys*-like infections in water buffalo in Thailand

Two species of *Anaplasma* (*A. marginale* and *A. platys*-like) were detected in the blood of Thai water buffalo in the present study. Because there has not been a previous report of *A. platys* infection in buffalo in Thailand, we re-confirmed this result by conventional Polymerase Chain Reaction (cPCR) amplification and sequencing of the heat shock protein (groEL) gene. Homology searching of the obtained sequences against the GenBank™ database using the BLASTn algorithm revealed that the three obtained sequences had a high percentage similarity (98.36–99.62%) to *A. platys* sequences (accession nos. MH716429–36) (Table 3).

**Table 3:** Species identification based on BLASTn search results

Gene target	No. of sequenced samples	Sequences with a significant alignment	Reference sequence	No. of bp matched (bp)	% Similarity
*Anaplasma* spp. 16S rRNA	40	*Anaplasma marginale*: 37/40	KT264188	1067/1123 – 1121/1123	95.05–99.83%
		*Anaplasma platys*: 3/40	EF139459	1119/1124 – 1121/1124	99.53–99.76%
*A. platys* groEL	3	*Anaplasma platys:* 3/3	MH716435	764/777 – 774/777	98.36–99.62%

In order to determine the genetic relationship of the tick-borne rickettsial organisms among the different buffalo in these Thai populations, pairwise nucleotide identity analysis of the 16S ribosomal RNA (16S rRNA) sequences was conducted. For *A. marginale*, the 16S rRNA gene showed a percentage identity match ranging from 98.4–100% (Table 4), while the three *A. platys* 16S rRNA sequences were 100% identical. Comparing these three *A. platys* 16S rRNA sequences in buffalo in Thailand with those from other countries, they were more similar to the *A. platys* sequences in China, South Africa, and Thailand (99.7%) than to those in Venezuela (99.5%) and Vietnam (99.3%) (Table 5).

**Table 4:** Pairwise nucleotide identity matrix of within-population *A. marginale* from Thailand and global isolates based on the 16S rRNA gene

	Identity (%)										
Isolate	1	2	3	4	5	6	7	8	9	10	11
1. North China (HM538192)	100.0										
2. Central China (AJ633048)	99.8	100.0									
3. South China (DQ341370)	100.0	99.8	100.0								
4. Southwest China (HM538193)	99.0	98.8	99.0	100.0							
5. Southeastern U.S.A. (AF311303)	100.0	99.8	100.0	99.0	100.0						
6. Uganda (KU686794)	99.9	99.7	99.9	98.9	99.9	100.0					
7. Central Philippines (JQ839012)	100.0	99.8	100.0	99.0	100.0	99.9	100.0				
8. Thailand (KT264188)	100.0	99.8	100.0	99.0	100.0	99.9	100.0	100.0			
9. Thailand (MN658600) Haplotype 1	100.0	99.8	100.0	99.0	100.0	99.9	100.0	100.0	100.0		
10. Thailand (MN658608) Haplotype 2	99.4	99.2	99.4	**98.4**	99.4	99.5	99.4	99.4	99.4	100.0	
11. Thailand (MN658622) Haplotype 3	99.9	99.7	99.9	98.9	99.9	99.8	99.9	99.9	99.9	99.3	100.0

Sequence pair with the lowest % identity is in bold. Note that representative sequences that originated from China, USA, Uganda, and the Philippines were used. Haplotype 1 (*n* = 22); Haplotype 2 (*n* = 9); and Haplotype 3 (n = 6)

**Table 5:** Pairwise nucleotide identity matrix of within population *A. platys*-like from buffalo in Thailand and worldwide isolates based on the 16S rRNA gene

	Identity (%)							
Isolate	1	2	3	4	5	6	7	8
1. China (MH762081) /Tick	100.0							
2. Vietnam (MH686048) /Cattle	99.5	100.0						
3. South Africa (MK814449) /Cattle	100.0	99.5	100.0					
4. Venezuela (AF399917) /Dog	99.7	**99.3**	99.7	100.0				
5. Thailand (EF139459) /Dog	100.0	99.5	100.0	99.7	100.0			
6. Thailand (MN658639) /Water buffalo	99.7	**99.3**	99.7	99.5	99.7	100.0		
7. Thailand (MN658640) /Water buffalo	99.7	**99.3**	99.7	99.5	99.7	100.0	100.0	
8. Thailand (MN658641) /Water buffalo	99.7	**99.3**	99.7	99.5	99.7	100.0	100.0	100.0

Sequence pair with the lowest % identity is in bold. Note that representative sequences that originated from China, Vietnam, South Africa, and Venezuela were used

We obtained a total of 43 sequences, 37 for the 16S rRNA gene of *A. marginale* (1123 bp length), and three each for the 16S rRNA (1124 bp) and groEL (777 bp) genes of *A. platys*. Three different haplotypes of *A. marginale* in the present study were identified using the DnaSP software, and were comprised of haplotype 1 (accession nos. MN658600–07, MN658609, MN658612–21, MN658625, and MN658632–33); haplotype 2 (accession nos. MN658608, MN658610–11, MN658623–24, MN658626, and MN658634–36); and haplotype 3 (accession nos. MN658622 and MN658627–31). When compared to *A. marginale* isolates from other geographic regions, the haplotype network showed that there were six haplotypes among 11 countries (Additional file 2: Figure S2). Furthermore, most of the *A. marginale* isolates obtained from other countries were classified as haplotype 1, the same as the dominant haplotype found in Thailand, except for haplotypes 4 and 6 present in China and haplotype 5 in India.

**Fig. 1:**
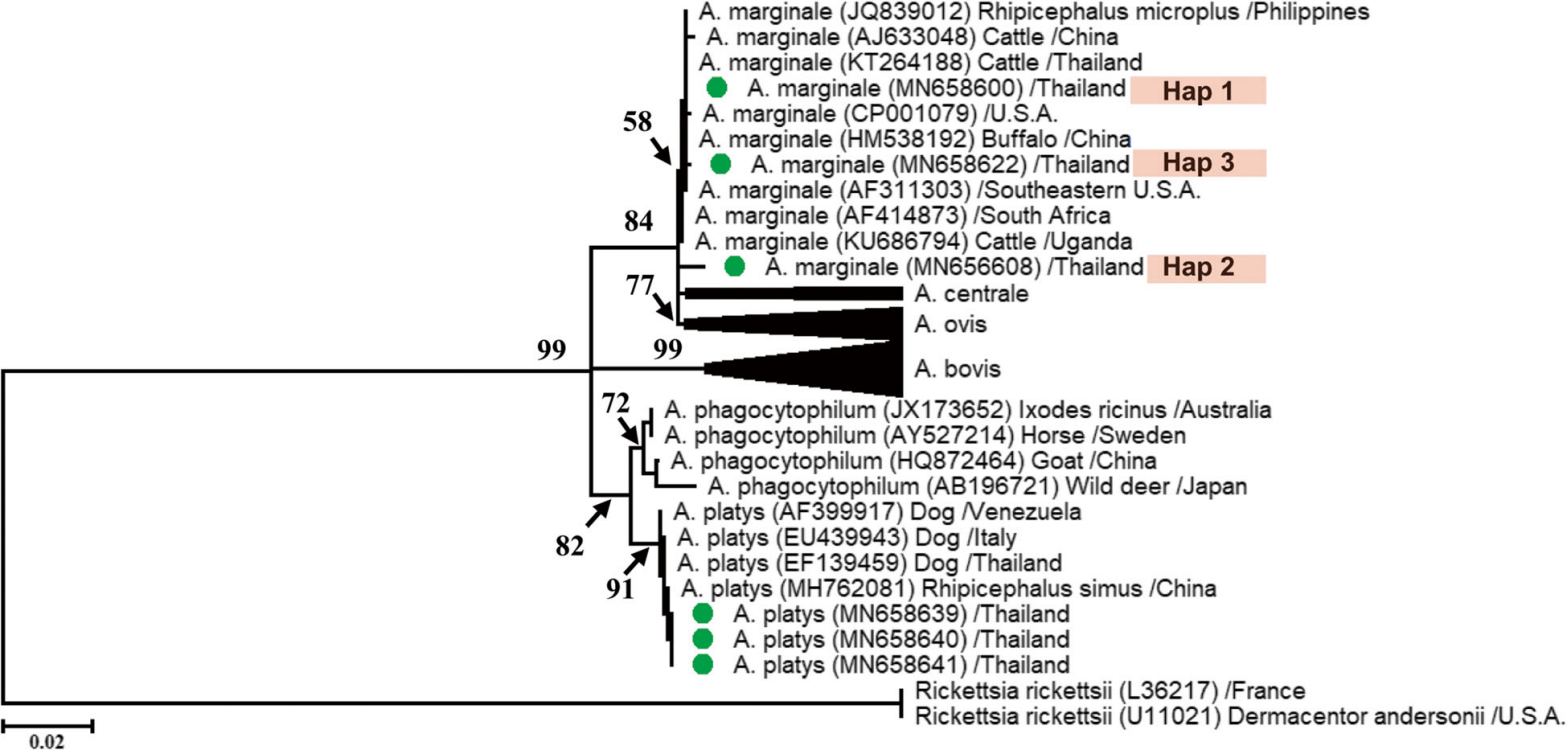
A ML phylogenetic tree based on the 16S rRNA gene fragment (1124 bp) of *A. marginale* and *A. platys-*like using the Kimura 2 parameter model. Taxa with green circles are from the present study, while BS values greater than 50% are shown in the figure. The two *Rickettsia rickettsii* sequences from *Dermacentor andersonii* (host tick) in France and the USA, respectively, were used as outgroups

One representative of each of *A. marginale* haplotype together with three 16S rRNA *A. platys* isolates were chosen to construct the phylogenetic tree using the MEGA version 10.0.5 software. The maximum likelihood (ML) tree of *Anaplasma* spp. constructed with the 16S rRNA sequences indicated that the *A. marginale* and *A. platys* found in Thai buffalo isolates clustered into two different clades (Fig. 1). The three haplotypes of *A. marginale* isolates were relatively similar to each other and placed in a clade that was closely related to *A. marginale* in buffalo in China (accession no. HM538192); cattle in Thailand (accession no. KT264188), Uganda (accession no. KU686794), and China (accession no. AJ633048); and ticks in the Philippines (accession no. JQ839012), South Africa (accession no. AF414873), USA (accession no. CP001079), and Southeastern USA (accession no. AF311303).

**Fig. 2:**
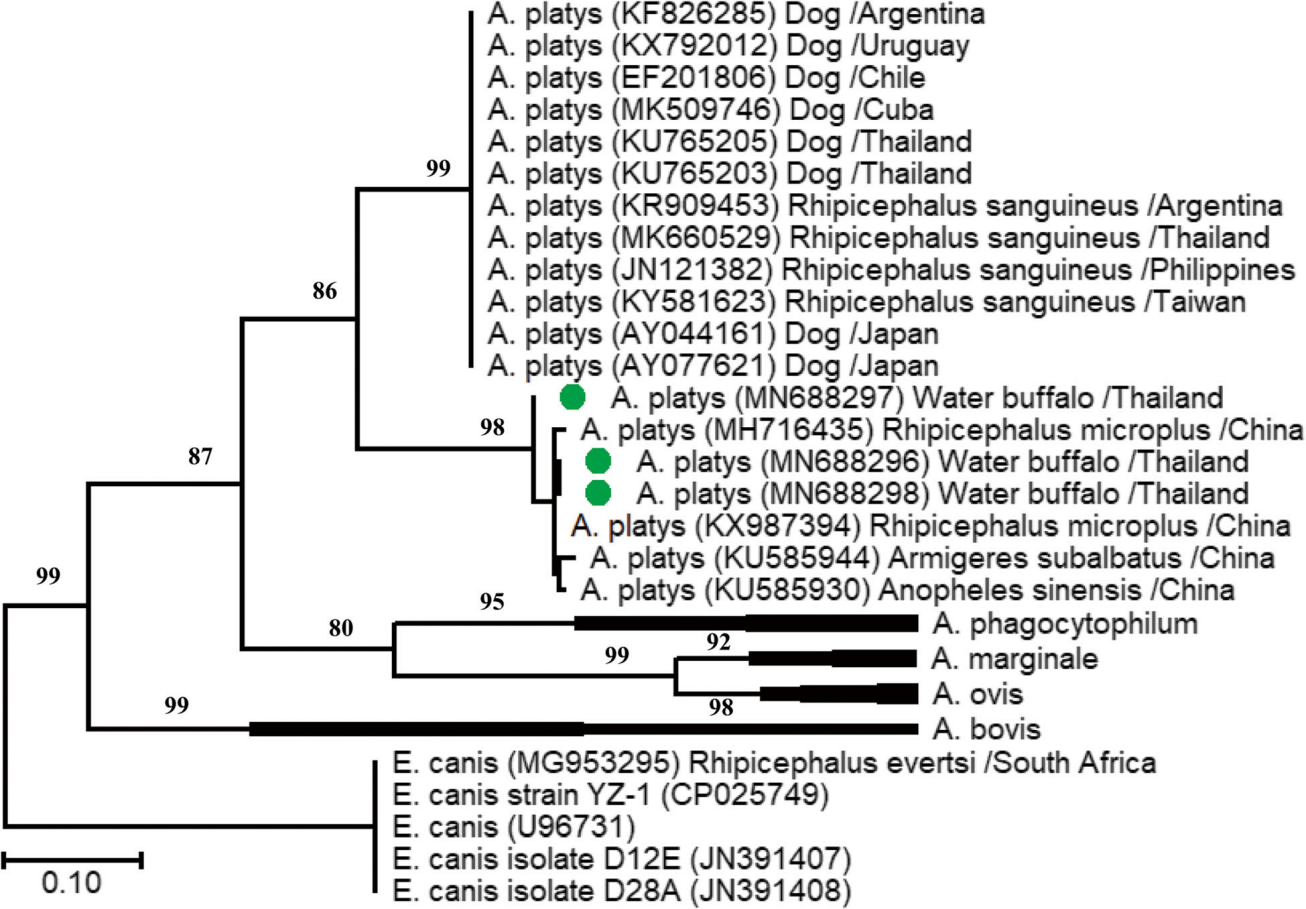
A ML phylogenetic tree of *A. platys-*like inferred from the groEL gene fragment (777 bp), using the Tamura 3-parameter model. The groEL sequences of *E. canis* were used as the outgroups. Taxa with green circles are from the present study, while BS values greater than 50% are shown in the figure

For the *A. platys* groEL gene-based ML tree, all three sequences fell into one cluster corresponding to the *A. platys* recovered from tropical cattle ticks (accession nos. MH716435 and KX987394) and mosquitoes (accession nos. KU585930 and KU585944) in China (Fig. 2). It is important to note that *A. platys* sequences from Thai buffalo (this study) formed a distinct branch separate from the *A. platys* previously recovered from dogs in Thailand (accession nos. KU765203 and KU765205), Japan (accession nos. AY044161 and AY077621), Argentina (accession no. KF826285), Cuba (accession no. MK509746), Uruguay (accession no. KX792012), and Chile (accession no. EF201806); and brown dog ticks in Thailand (accession no. MK660529), Argentina (accession no. KR909453), Philippines (accession no. JN121382), and Taiwan (accession no. KY581623).

### Within population nucleotide diversity of *A. marginale* and *A. platys*-like from buffalo in Thailand

The nucleotide diversity of *A. marginale* 16S rRNA and *A. platys* groEL are summarized in Table 6. The three *A. platys* isolates were shown to have identical 16S rRNA gene sequences over the 1124 bp aligned nucleotide positions with no segregating sites and so only one haplotype.

**Table 6:** Nucleotide diversity of *A. marginale* and *A. platys*-like sequences obtained from buffalo isolates in Thailand

Gene target	(bp)	N	S	H	Hd	π
16S rRNA (*A. marginale*)	1123	37	29	18	0.826	0.00586
groEL (*A. platys*)	777	3	5	2	0.667	0.00429

*N* number of sequences analyzed, *S* number of polymorphic (segregated) sites, *H* number of haplotypes, *Hd* Haplotype diversity; *π* nucleotide diversity (Pi)

### Synonymous nucleotide substitutions in the *A. platys*-like groEL gene from water buffalo isolates in Thailand

Among the three groEL gene sequences from *A. platys*-like samples isolated from water buffalo in Thailand, two sequences (accession nos. MN688296 and MN688298) were identical. For the third, there were five polymorphic nucleotides compared to the other two sequences. Individually, these nucleotide substitutions observed in isolate THBuff16–83 (accession no. MN688297) were also found in other sequences in the GenBank™ database. At position 162, *A. platys*-like from Thai buffalo (G162T) was the same as those recovered from the tropical cattle tick in China (accession no. KX987394) and *Ehrlichia canis* from *Rhipicephalus evertsi* in South Africa (accession no. MG953295). Nucleotide position 583 in *A. platys* from Thai buffalo (T583C) had the same nucleotide as *A. platys* recovered from the brown dog tick (*R. sanguineus*) in Argentina, Thailand, the Philippines, and Taiwan; and from dogs in Argentina, Uruguay, Cuba, Thailand, and Japan. Interestingly, position 687 of *A. platys*-like in the Thai buffalo (G/T687A) shared the same nucleotide to *A. platys* detected from mosquitoes in China (accession nos. KU585930 and KU585944) but was different from all the other sequences. The remaining two polymorphic sites, nucleotides 168 (A168G) and 696 (C696A), had the same substitutions as *E. canis* from *R. evertsi* in South Africa (accession no. MG953295) (Table 7). Although there were five substitutions among the three *A. platys*-like isolates from water buffalo in Thailand, they were all synonymous substitutions and so the deduced amino acid sequence of these groEL genes in the present study were identical among the three Thai isolates.

**Table 7:** Nucleotide substitutions of groEL gene among *A. platys*-like from buffalo isolates in Thailand and global isolates from different host origins

Accession no.	Host origin (Scientific name)	Country	Nucleotide position				
			162	168	583	687	696
MN688296*	Water buffalo *(Bubalus bubalis)*	Thailand	G	A	T	G	C
MN688297*	Water buffalo *(B. bubalis)*	Thailand	T	G	C	A	A
MN688298*	Water buffalo *(B. bubalis)*	Thailand	G	A	T	G	C
KU765205	Dog *(Canis lupus familiaris)*	Thailand	–	–	C	T	C
KU765203	Dog *(C. lupus familiaris)*	Thailand	–	–	C	T	C
AY044161	Dog *(C. lupus familiaris)*	Japan	G	A	C	T	C
AY077621	Dog *(C. lupus familiaris)*	Japan	G	A	C	T	C
KF826285	Dog *(C. lupus familiaris)*	Argentina	–	–	C	T	C
KX792012	Dog *(C. lupus familiaris)*	Uruguay	–	–	C	T	C
MK509746	Dog *(C. lupus familiaris)*	Cuba	–	–	C	T	C
KP027339	Dog *(C. lupus familiaris)*	Philippines	G	A	–	–	–
KR909453	Tick *(Rhipicephalus sanguineus)*	Argentina	–	–	C	T	C
MK660529	Tick *(R. sanguineus)*	Thailand	–	–	C	T	C
JN121382	Tick *(R. sanguineus)*	Philippines	G	A	C	T	C
KY581623	Tick *(R. sanguineus)*	Taiwan	G	A	C	T	C
KX987394	Tick *(Rhipicephalus microplus)*	China	T	A	T	G	C
MH716435	Tick *(R. microplus)*	China	G	A	T	G	C
KU585930	Mosquito *(Anopheles sinensis)*	China	G	A	T	A	C
KU585944	Mosquito *(Armigeres subalbatus)*	China	G	A	T	A	C
MG953295 *(E. canis)*	Tick *(Rhipicephalus evertsi)*	South Africa	T	G	T	T	A

Asterisk indicates sequences obtained in the present study. Nucleotide positions of the groEL gene from *A. platys* were numbered after KU585930, KU585944, JN121382, KX987394, MH716435, KY581623, AY044161, and AY077621. Dashed line indicates that the nucleotides in those positions were missing since the deposited sequences did not cover that region

## Discussion

Water buffalo frequently show less severe clinical anaplasmosis symptoms than those seen in infected cattle under the same environmental conditions, which is likely to be at least partly due to their breed resistance [2]. Although clinical anaplasmosis is most notable in cattle, water buffalo can become persistently infected and harbor a sub-clinical disease [18]. Whilst clinical anaplasmosis has rarely been detected in buffalo in Thailand, the possibility for these animals to harbor rickettsia has not been widely investigated. The two dominant tick species commonly found in Thailand in cattle and dogs are *Rhipicephalus microplus* and *R. sanguineus*, respectively, and so they could be vectors.

This study reported the molecular detection and identification of rickettsial organisms in water buffalo from different geographic provinces. The prevalence of rickettsial organisms varied among the eight provinces at a range of 6–67.6% with an overall average prevalence rate of 41%, as based on nPCR amplification with our newly designed primers. It is also important to note that with the cPCR assays using the *Anaplasmataceae*-specific primers as previously described [19], an overall lower average prevalence rate (34.42%) was obtained, but remain 83.96% agreement with our nPCR. This could be partly explained by the different sensitivities between the cPCR and nPCR or by a storage effect on the DNA templates, since the cPCR amplifications with the *Anaplasmataceae*-specific primers were performed several months after the nPCR amplifications.

This high prevalence of buffalo positive for infection with rickettsial organisms was similar to those observed previously in Cuba (52% [20]) and Mozambique (72.2% [21]), but was higher than those reported in the Philippines (29% [22]), Malaysia (21.8% [23]), India (18.33% [24]), South Africa (17.3% [25]), Pakistan (14.73% [17]), Columbia (13.1% [26]), and northeast Thailand (8% [11]). In this study, the highest anaplasmosis prevalence was found in Southern Thailand (Phatthalung), while the lowest was at Northeastern Thailand (Nong Bua Lamphu). Note that the Uthai Thani, Amnat Charoen, Nong Bua Lamphu, Phatthalung, and Chachoengsao provinces in Thailand were surveyed for the first time in this study.

The different prevalence of *A. marginale* in buffalo among regions in Cuba has been reported to be dependent on environmental factors, including the tick population, season, and management system, in each farm [20]. For Thailand, one reason that can explain this variation is the diverse weather between the different regions of the country. Southern Thailand is humid with a high temperature all year-round, and so ticks are more likely to be present and at higher densities than in other areas (https://www.tmd.go.th/). In this study, female buffalo had significantly higher infection rate than male ones. The possible explanation for this is probably because the different number of male and female buffalo blood samples were collected. The number of female buffalo was approximately four times higher than the male ones. Nevertheless, this result was contradicted to the previous findings in Pakistan, where male buffalo had a higher disease prevalence (26.25%) than females (16.62%), although this was from a larger sample size of 118 males and 617 females [17]. In addition, the age of the water buffalo also plays a role in disease susceptibility. Young animals are more likely to be susceptible to *A. marginale* infection compared to adult cattle, because their softer skin facilitates the mouth-part penetration of the vector, making them the preferred host of ticks [27]. Thus, the prevalence of tick-borne rickettsial organisms in buffalo, which varied from region to region in Thailand, could be associated with the host age, gender, and breed, plus the tick density according to the season and animal husbandry or management of each farm. *Ehrlichia* spp., *Neorickettsia* spp., and *Wolbachia* spp. were not detected in any of the sequenced samples in the present study. The only reported *Ehrlichia* spp. in cattle, *E. ruminantium*, a causative agent of heartwater (cowdriosis), appears to be restricted to African buffalo in Northern Botswana [28], and to cattle in Mozambique [29], and China [30].

In the present study, PCR amplification with the *A. marginale* species-specific primers showed that the majority of detected rickettsial organisms were *A. marginale* (74.87% of all rickettsial pathogens-positive samples), which was in agreement with previous reports from Thailand [11] and the Philippines [22]. However, it is important to note that only 37 of these ‘*A. marginale’* amplified sequences were confirmed for species designation (i.e. primer specificity for that species) by sequencing. The three remaining rickettsial samples were *A. platys*-like and were only found in one province (Lampang). Thus, *A. platys*-like infection in water buffalo in Thailand is not as widely distributed as that for *A. marginale*. For the *A. platys*-like species, the isolates found in water buffalo in Thailand all belonged to the *A. platys* group and were closely related to the *A. phagocytophilum* group and placed separately to *A. marginale.* This result was in agreement with the finding in Mozambique, where *A. platys* were related to *A. phagocytophilum* with a genetic divergence of 0.8% [21]. A similar finding was also observed in previous studies in Vietnam [8] and Algeria [31], while *A. platys*-like infections in Tunisian cattle, goats, and sheep [32], and camels [33] confirmed that *A. platys* is not dog-specific. Indeed, whilst *A. marginale* is mainly responsible for anaplasmosis in cattle and buffalo, *A. platys* is also known to infect dogs, cattle [8], and humans [5–7].

The buffalo blood samples in this study were collected from buffalo farms, where dogs were present in the buffalo stalls. The brown dog tick, *R. sanguineus*, is believed to transmit *A. platys* (based upon the frequent finding of DNA from *A. platys* in the tick), but we were not able to confirm it in the present study. Moreover, the groEL sequences of these three *A. platys*-like isolates were clustered in the same clade as the *A. platys* from the tick *R. microplus*, and the mosquitoes *Anopheles sinensis* and *Armigeres subalbatus*. Therefore, these tick and mosquitoes cannot be ruled out as potential vectors for *A. platys* transmission.

The 16S rRNA gene is a common target for pathogen detection and species identification and is also used to infer phylogenetic relationships. However, this gene is relatively highly conserved, as demonstrated by its relatively low level of polymorphism and genetic diversity compared to other gene targets, such as the outer membrane protein msp1α [34–36], and msp4 [14, 34]. Indeed, although msp1α, msp4, and msp5 are relatively conserved genes, they were shown to be useful for phylogenetic analysis among different geographic isolates of *A. marginale* strains [35]. Thus, the low intra-population level of polymorphism and genetic diversity of *A. marginale* and *A. platys*-like isolated from water buffalo in Thailand in this study may under-predict their actual level of genetic variation. Regardless, these results are consistent with those of previous observations that *A. marginale* 16S rRNA sequences show genetic homogeneity within populations, and suggest that besides the 16S rRNA gene, the outer membrane protein and heat shock protein genes might be ideal targets for the detection, identification, and genetic characterization of rickettsial organisms in different geographic locations.

The limitation of this study is that the blood samples were collected at a single time point of the year. Therefore, the present results might not totally represent the year-round observation regarding the infection rate in buffalo. Sample collections should be carried out in every season within a year if possible, to determine and assess the effects of different seasons on prevalence and infection rate. Furthermore, identification of tick is also important for vector management, disease control and prevention. Thus, these issues are of interest and should be conducted in the future.

## Conclusion

Our findings suggest that water buffalo may play an important role as a reservoir host of *A. marginale*. The ML phylogenetic analysis of *A. platys*-like species indicated that the isolates found in Thai buffalo were more closely related to the *A. platys* recovered from cattle ticks and mosquitoes than from dogs and brown dog ticks. The present study is the first report of *A. platys*-like species in water buffalo in Thailand, which is of importance as *A. platys* has previously been reported as a zoonotic species [5–7]. Therefore, its potential as a tick-borne pathogen from animals to humans should not be overlooked.

## Methods

### Study sites and blood collections

**Fig. 3:**
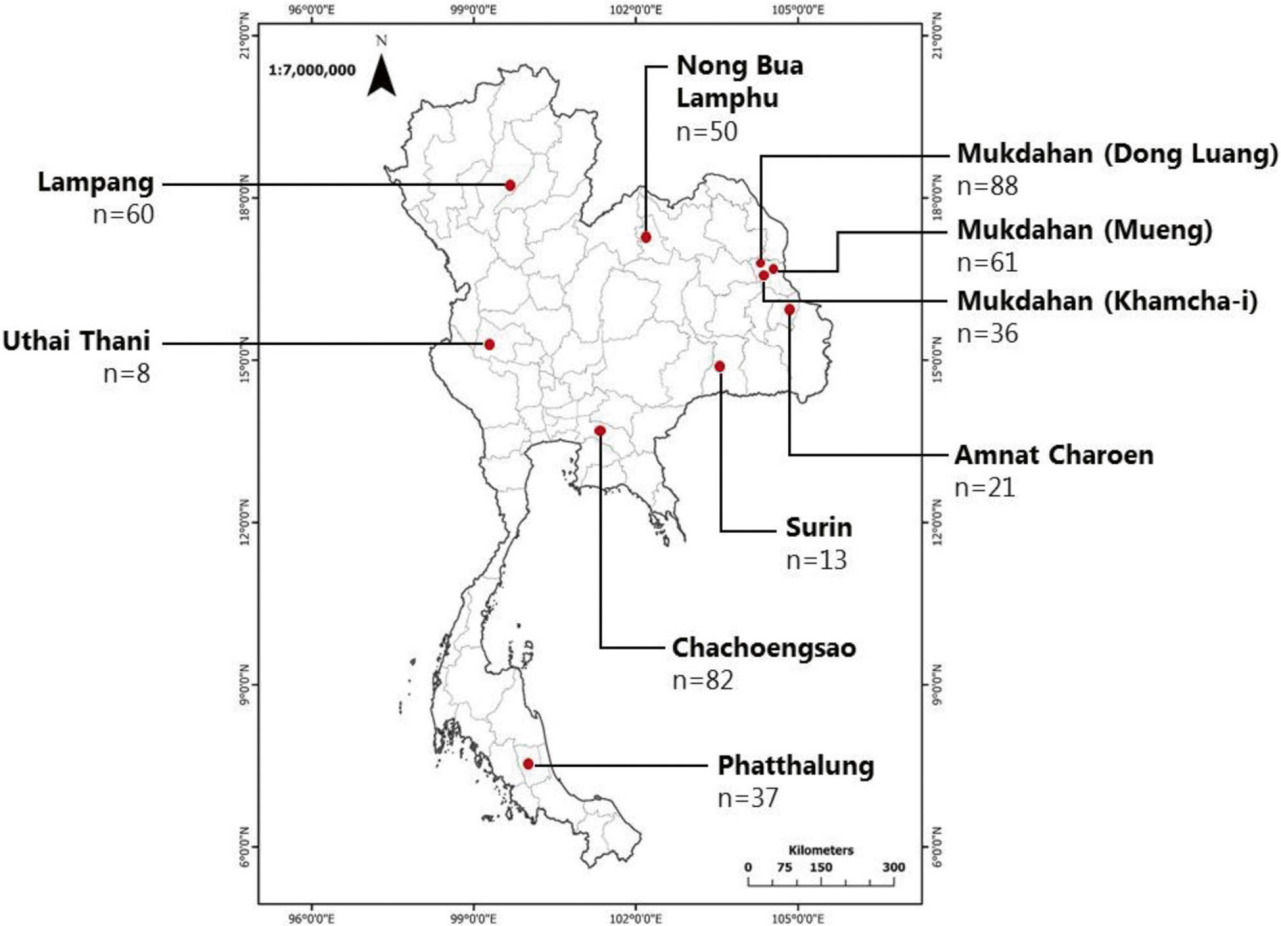
Map showing the sampling areas in Thailand and the number of samples from each location. The map was drawn using ArcGIS version 10.2

A cross-sectional study of tick-borne rickettsial organism infection was conducted during January 2015 to June 2018. Buffalo blood samples were collected from eight geographic sites (provinces) within Thailand (Fig. 3) located in the Northern and Northeastern provinces of Lampang (*n* = 60), Nong Bua Lamphu (*n* = 50), Mukdahan (*n* = 185), Amnat Charoen (*n* = 21), and Surin (*n* = 13); in the Central region province of Uthai Thani (*n* = 8); in the Eastern province of Chachoengsao (*n* = 82); and in the Southern province of Phatthalung (*n* = 37). Animal restraint and blood collections were performed as previously described [37, 38]. Blood was collected into acid citrate dextrose-anticoagulant tubes, transported to the laboratory and used for subsequent DNA extraction.

### DNA extractions

Genomic DNA was extracted from 1.5 mL of blood using a NucleoSpin® Blood (Macherry-Nagel, Germany) following the manufacturer’s instructions, except that the final elution buffer volume was reduced to 50 μL. The extracted DNA was then stored at − 20 °C until subsequent PCR amplification.

### Screening for rickettsial organisms by PCR

Oligonucleotide primers for the detection of rickettsial organisms by PCR amplification of the 16S rRNA gene were designed (Additional file 1: Figure S1). A nPCR assay was used for *A. marginale* detection, targeting its 16S ribosomal RNA gene, while cPCR was used for re-confirmation of *A. platys* using primers to specifically amplify the groEL gene (Additional file 2: Table S1). DNA samples were re-confirmed by *Anaplasmataceae*-specific primers EHR16SR (5′-TAG-CAC-TCA-TCG-TTT-ACA-GC-3′) and EHR16SD (5′-GGT-ACC-YAC-AGA-AGA-AGT-CC-3′) [19]. Other species-specific primers targeting the major surface protein 2 gene of *A. marginale*, MSP2-F (5′-CAC-CAT-GAG-TGC-TGT-AAG-TAA-TAG-GAA-GC-3′) and MSP2-R (5′-CTA-GAA-GGC-AAA-CCT-AAC-ACC-CAA-CTC-3′) were used to identify the *A. marginale* in rickettsial organism-positive samples in this study [39]. All *A. platys*-like species detected in buffalo were also verified using the species-specific primers PLATYS-F (5′-AAG-TCG-AAC-GGA-TTT-TTG-TC-3′) and PLATYS-R (5′-CTT-TAA-CTT-ACC-GAA-CC-3′) [40]. The PCR reactions were performed in a final volume of 12.5 μL consisting of 6.25 μL of 2X PCR buffer KOD FX Neo, 2.5 μL of dNTPs (0.4 mM each), 0.375 μL of each primer (10 pmol/ μL), 0.25 μL of KOD FX Neo DNA polymerase (Toyobo, Japan), 1 μL of the extracted DNA template (ca. 15–20 ng), and 1.75 μL of sterile distilled water. The PCR thermocycling condition to screen for rickettsial organisms was comprised of 94 °C for 2 min, followed by 40 cycles of 98 °C for 10 s, 55 °C for 30 s, and 68 °C for 90 s, and then a final 68 °C for 5 min. Genomic DNA of *A. marginale* isolate AmCU01, *Ehrlichia* spp., *Wolbachia* spp., *A. platys*, and *A. bovis,* which were previously confirmed by Wattanamethanont et al. [16] (GenBank™ accession nos. KT264188, KJ410253, KM404238, KU500914, and KP314253, respectively), were used as the positive controls, while non-template sterile distilled water was used as a negative control.

After the first round of the cPCR, the primary cPCR products were diluted 1:10 (v/v) with distilled water and used as the DNA template for the second round nPCR. The nPCR amplifications were performed under the same thermocycling condition as the primary cPCR above except for the primers. The *A. platys*-like isolates obtained from the 16S rRNA gene sequencing were subsequently confirmed by cPCR amplification and sequencing of the groEL gene, performed as above except for the primers (Table S1) and the annealing temperature was 58 °C for 30 s. All PCR reactions were performed in an Axygen® MaxyGene II Thermal Cycler (Life Sciences, USA). Gel electrophoresis was performed at 100 V and 400 mA for 45 min in 1.5% (w/v) agarose gel with 0.5X TAE buffer. The gel was stained with ethidium bromide and the PCR products were visualized under a UV transilluminator.

### Sequencing preparation

Approximately 20% of the rickettsial organism-positive samples in each province were chosen and scaled up to 25 μL of PCR product before being prepared for sequencing. Those PCR products without non-specific bands were treated with a 10-fold dilution of ExoSAP-IT™ (USB Corporation, USA) according to the manufacturer’s instruction to digest the remaining single-stranded DNA at 37 °C for 15 min to degrade the residual primers and nucleotides and then at 80 °C for 15 min to inactivate the ExoSAP-IT™ reagent. The ExoSAP-IT™-treated PCR products were then directly sequenced in both directions using the same primers as the PCR amplification. For confirmation of samples designated from the obtained 16S rRNA sequences as *A. platys-*like species, a new 50-μL PCR reaction containing fresh DNA template was amplified with primers targeting the groEL gene. Amplicon bands were extracted from the resolved agarose gel using NucleoSpin® Gel and PCR Clean-up (Macherry-Nagel, Germany) kits following the manufacturer’s recommendations, and then sent for commercial sequencing using the same forward and reverse primers as in the PCR assay.

### DNA sequence analyses and assessment of the host-pathogen interaction

The obtained 16S rRNA and groEL sequences were visually checked and manually corrected where necessary using the BioEdit version 7.0.5.3 software (freely available at www.mbio.ncsu.edu). Any singleton mutation was confirmed from the sequencing results of at least two independent PCR products. Ambiguous sequences were excluded from further analyses. To determine the species of rickettsial organisms, the 16S rRNA nucleotide sequences obtained from the sequencing results were compared and matched to species-annotated sequences in the GenBank™ database using the BLASTn search algorithm (http://blast.ncbi.nlm.nih.gov/Blast.cgi). Haplotype analysis and genetic diversity of the nucleotide sequence data was assessed using the DnaSP version 6.12.01 software (available at www.ub.edu/dnasp). Haplotype network was created by Median Joining Network using Population Analysis with Reticulate Trees (PopART) version 1.7 (available at http://popart.otago.ac.nz/downloads.shtml).

Phylogenetic trees were constructed based on the lowest Bayesian Information Criterion score by the maximum likelihood (ML) method as implemented in the MEGA X software. Support for each node was assessed by bootstrapping (BS) using 1000 replicates. Nucleotide sequences in the present study have been deposited in the GenBank™ database under accession numbers: MN658600–36 for *A. marginale* 16S rRNA, MN658639–41 for *A. platys* 16S rRNA, and MN688296–8 for *A. platys* groEL. Reference sequences of *A. marginale/ A. centrale, A. platys, A. bovis, A. ovis,* and *A. phagocytophilum* for constructing the phylogenetic trees were retrieved from the GenBank™ database and are listed in Additional file 3: Table S2.

### Statistical analysis

Data analysis was performed using the SPSS version 22 software. The interaction between pathogen and host (buffalo) age or gender was analyzed using Pearson correlation coefficient and *p*-values ≤0.05 were deemed significant. The 95% confidence intervals (CI) for the OR were calculated based on the Mantel Haenszel distribution. The univariate general linear model was used to recheck the association between pathogen and host.

## Supplementary information

**Additional file 1: Figure S1** Clustal Omega sequence alignment of the 16S rRNA and groEL genes depicting the primer design.

**Additional file 2: Figure S2** Median Joining Network of *A. marginale* based on 16S rRNA haplotype among Thailand and other countries.

**Additional file 3: Table S1** Oligonucleotide primers used in this study. **Table S2** Reference sequences of 16S rRNA and groEL genes from global isolates included in the phylogenetic analyses.

## Data Availability

All data generated or analyzed during this study are included in this manuscript.

## Abbreviations

cPCR: conventional Polymerase Chain Reaction
nPCR: nested Polymerase Chain Reaction
bp: base pair
ML: Maximum likelihood
BS: Bootstrapping
16S rRNA: 16S ribosomal ribonucleic acid
OR: Odds Ratio
groEL: heat shock protein groEL
TAE: Tris-acetate-EDTA
N: number of sequences analyzed
S: number of polymorphic sites
H: number of haplotypes
Hd: Haplotype diversity
π: nucleotide diversity

## References

[1] Indramangala J. Buffalo development in Thailand. http://breedplan.une.edu.au/thailand/forms_docs/buffalo_acr.pdf (2002). Accessed 24 March 2020..

[2] Rajput ZI, Hu S-H, Arijo AG, Habib M, Khalid M (2005). Comparative study of *Anaplasma* parasites in tick carrying buffalo and cattle. J Zhejiang Univ Sci B.

[3] Dreher UM, de la Fuente J, Hofmann-Lehmann R, Meli ML, Pusterla N, Kocan K, Woldehiwet Z, Braun U, Regula G, Staerk KDC, Lutz H (2005). Serologic cross-reactivity between *Anaplasma marginale* and *Anaplasma phagocytophilum*. Clin Diagn Lab Immunol.

[4] Stuen S, Granquist EG, Silaghi C (2013). *Anaplasma phagocytophilum* – a widespread multi-host pathogen with highly adaptive strategies. Front Cell Infect Microbiol.

[5] Maggi RG, Mascarelli PE, Havenga LN, Naidoo V, Breitschwerdt EB (2013). Co-infection with *Anaplasma platys*, *Bartonella henselae* and *Candidatus Mycoplasma haematoparvum* in a veterinarian. Parasit Vectors.

[6] Arraga-Alvarado CM, Qurollo BA, Parra OC, Berrueta MA, Hegarty BC, Breitschwerdt EB (2014). Molecular evidence of *Anaplasma platys* infection in two women from Venezuela. Am J Trop Med Hyg.

[7] Breitschwerdt EB, Hegarty BC, Qurollo BA, Saito TB, Maggi RG, Blanton LS (2014). Intravascular persistence of *Anaplasma platys*, *Ehrlichia chaffeensis*, and *Ehrlichia ewingii* DNA in the blood of a dog and two family members. Parasit Vectors.

[8] Chien NTH, Nguyen TL, Bui KL, Nguyen TV, Le TH (2019). *Anaplasma marginale* and *A. platys* characterized from dairy and indigenous cattle and dogs in northern Vietnam. Korean J Parasitol.

[9] Kocan K, de la Fuente J, Blouin EF, Coetzee JF, Ewing SA (2010). The natural history of *Anaplasma marginale*. Vet Parasitol.

[10] Vatsya S, Kumar RR, Singh VS, Arunraj MR (2013). *Anaplasma marginale* infection in a buffalo: a case report. Vet Res Int.

[11] Saetiew N, Simking P, Inpankaew T, Wongpanit K, Kamyingkird K, Wongnakphet S, Stich RW, Jittapalapong S (2015). Prevalence and genetic diversity of *Anaplasma marginale* infections in water buffalo in Northeast Thailand. J Trop Med Parasitol.

[12] Swift BL, Paumer RJ. Vertical transmission of *Anaplasma marginale* in cattle. Theriogenology. 1976;6(5):515–21.

[13] Kaewthamasorn M, Wongsamee S (2006). A preliminary survey of gastrointestinal and haemoparasites of beef cattle in the tropical livestock farming system in Nan Province, northern Thailand. Parasitol Res.

[14] Jirapattharasate C, Adjou Moumouni PF, Cao S, Iguchi A, Liu M, Wang G (2017). Molecular detection and genetic diversity of bovine *Babesia* spp., *Theileria orientalis*, and *Anaplasma marginale* in beef cattle in Thailand. Parasitol Res.

[15] Sumrandee C, Baimai V, Trinachartvanit W, Ahantarig A (2016). Molecular detection of *Rickettsia*, *Anaplasma*, *Coxiella* and *Francisella* bacteria in ticks collected from *Artiodactyla* in Thailand. Ticks Tick-borne Dis..

[16] Wattanamethanont J, Kaewthamasorn M, Tiawsirisup S (2018). Natural infection of questing ixodid ticks with protozoa and bacteria in Chonburi Province, Thailand. Ticks Tick-borne Dis.

[17] Farooqi SH, Ijaz M, Rashid MI, Nabi H, Islam S, Aqib AI (2018). et al. Molecular epidemiology of bovine anaplasmosis in Khyber Pakhtunkhwa, Pakistan. Trop Anim Health Prod.

[18] Kuttler K (1984). *Anaplasma* infections in wild and domestic ruminants: a review. J Wildl Dis.

[19] Parola P, Roux V, Camicas J-L, Baradji I, Brouqui P, Raoult D (2000). Detection of *Ehrlichiae* in African ticks by PCR. Trans R Soc Trop Med Hyg.

[20] Obregón D, González BC, de la Fuente J, Cabezas-Cruz A, Gonçalves LR, Matos C (2018). Molecular evidence of the reservoir competence of water buffalo (*Bubalus bubalis*) for *Anaplasma marginale* in Cuba. Vet Parasitol Reg Stud Reports.

[21] Machado RZ, Teixeira MM, Rodrigues AC, André MR, Gonçalves LR, da Silva JB (2016). Molecular diagnosis and genetic diversity of tick-borne Anaplasmataceae agents infecting the African buffalo *Syncerus caffer* from Marromeu Reserve in Mozambique. Parasit Vectors.

[22] Galon EMS, Adjou Moumouni PF, Ybañez RHD, Ringo AE, Efstratiou A, Lee S-H (2019). First molecular detection and characterization of tick-borne pathogens in water buffalo in Bohol, Philippines. Ticks Tick-borne Dis.

[23] Koh FX, Panchadcharam C, Sitam FT, Tay ST (2018). Molecular investigation of *Anaplasma* spp. in domestic and wildlife animals in peninsular Malaysia. Vet Parasitol Reg Stud Reports..

[24] Kumar N, Solanki JB, Varghese A, Jadav MM, Das B, Patel MD (2019). Molecular assessment of *Anaplasma marginale* in bovine and *Rhipicephalus (Boophilus) microplus* tick of endemic tribal belt of coastal South Gujarat, India. Acta Parasitol.

[25] Sisson D, Hufschmid J, Jolles A, Beechler B, Jabbar A (2017). Molecular characterisation of *Anaplasma* species from African buffalo (*Syncerus caffer*) in Kruger National Park, South Africa. Ticks Tick-borne Dis.

[26] Jaimes-Dueñez J, Triana-Chávez O, Mejía-Jaramillo AM (2018). Genetic, host and environmental factors associated with a high prevalence of *Anaplasma marginale*. Ticks Tick-borne Dis..

[27] Kabir MHB, Mondal MMH, Eliyas M, Mannan MA, Hashem MA, Debnath NC, Miazi OF, Mohiuddin C, Kashem MA, Islam MR, Elahi MF (2011). An epidemiological survey on investigation of tick infestation in cattle at Chittagong District, Bangladesh. Afr J Microbiol Res.

[28] Eygelaar D, Jori F, Mokopasetso M, Sibeko KP, Collins NE, Vorster I, Troskie M, Oosthuizen MC (2015). Tick-borne haemoparasites in African buffalo (*Syncerus caffer*) from two wildlife areas in northern Botswana. Parasite Vector.

[29] Matos CA, Gonçalves LR, de Souza Ramos IA, Mendes NS, Zanatto DCS, André MR (2019). Molecular detection and characterization of *Ehrlichia ruminantium* from cattle in Mozambique. Acta Trop.

[30] Guo H, Yin C, Galon EM, Du J, Gao Y, Adjou Moumouni PF (2018). Molecular survey and characterization of *Theileria annulata* and *Ehrlichia ruminantium* in cattle from Northwest China. Parasitol Int.

[31] Dahmani M, Davoust B, Benterki MS, Fenollar F, Raoult D, Mediannikov O (2015). Development of a new PCR-based assay to detect Anaplasmataceae and the first report of *Anaplasma phagocytophilum* and *Anaplasma platys* in cattle from Algeria. Comp Immunol Microbiol Infect Dis.

[32] Ben Said M, Belkahia H, El Mabrouk N, Saidani M, Alberti A, Zobba R (2017). *Anaplasma platys*-like strains in ruminants from Tunisia. Infect Genet Evol.

[33] Selmi R, Ben Said M, Dhibi M, Ben Yahia H, Messadi L (2019). Improving specific detection and updating phylogenetic data related to *Anaplasma platys*-like strains infecting camels (*Camelus dromedarius*) and their ticks. Ticks Tick-borne Dis.

[34] Ramos IA, Herrera HM, Mendes NS, Fernandes SD, Campos JB, Alves JV, Macedo GC, Machado RZ, André MR. Phylogeography of *msp4* genotypes of *Anaplasma marginale* in beef cattle from the Brazilian Pantanal. Rev Bras Parasitol Vet. 2019;28(3):451–7. 10.1590/S1984-29612019049 31390434

[35] Fedorina EA, Arkhipova AL, Kosovskiy GY, Kovalchuk SN (2019). Molecular survey and genetic characterization of *Anaplasma marginale* isolates in cattle from two regions of Russia. Ticks Tick-borne Dis..

[36] Fernandes SJ, Matos CA, Freschi CR, de Souza Ramos IA, Machado RZ, André MR (2019). Diversity of *Anaplasma* species in cattle in Mozambique. Ticks Tick-borne Dis..

[37] Templeton TJ, Asada M, Jiratanh M, Ishikawa SA, Tiawsirisup S, Sivakumar T, Namangala B, Takeda M, Mohkaew K, Ngamjituea S, Inoue N, Sugimoto C, Inagaki Y, Suzuki Y, Yokoyama N, Kaewthamasorn M, Kaneko O (2016). Ungulate malaria parasites. Sci Rep.

[38] Nguyen AHL, Tiawsirisup S, Kaewthamasorn M (2020). Low level of genetic diversity and high occurrence of vector-borne protozoa in water buffaloes in Thailand based on 18S ribosomal RNA and mitochondrial cytochrome b genes. Infect Genet Evol.

[39] Junsiri W, Watthanadirek A, Poolsawat N, Kaewmongkol S, Jittapalapong S, Chawengkirttikul R, Anuracpreeda P (2020). Molecular detection and genetic diversity of *Anaplasma marginale* based on the major surface protein genes in Thailand. Acta Trop.

[40] Inokuma H, Ohno K, Onishi T, Raoult D, Brouqui P (2001). Detection of Ehrlichial infection by PCR in dogs from Yamaguchi and Okinawa prefectures, Japan. Vet Med Sci.

