# Supplementary figures and images for: Molecular detection and genetic characterization of Anaplasma marginale and Anaplasma platys-like (Rickettsiales: Anaplasmataceae) in water buffalo from eight provinces of Thailand
Source: BMC Vet Res. 2020 Oct 8;16:380. doi: 10.1186/s12917-020-02585-z (PMC7542745; doi:10.1186/s12917-020-02585-z)

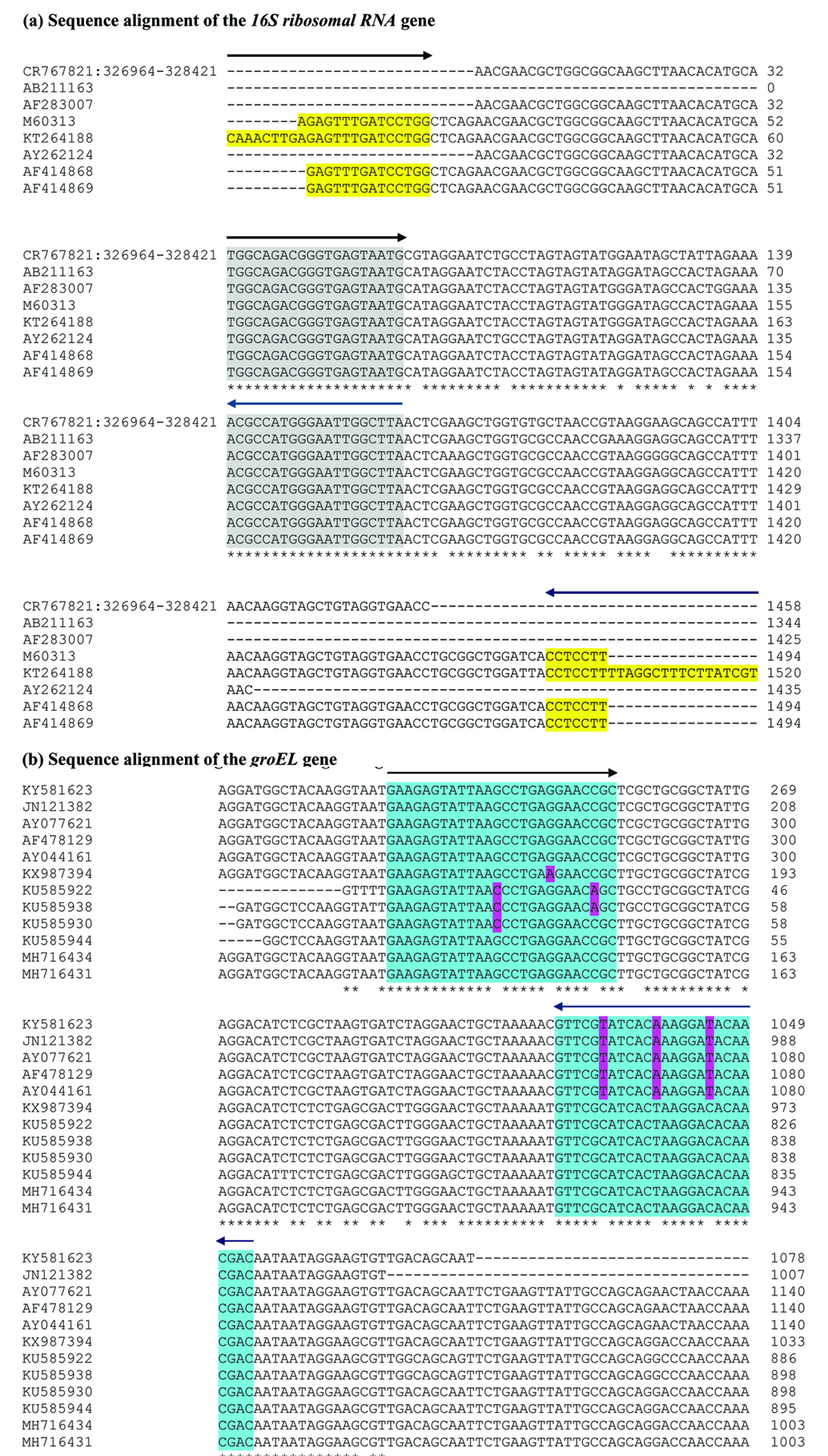

Supplement: Supplementary file 1 — Additional file 1: Figure S1 Clustal Omega sequence alignment of the 16S rRNA and groEL genes depicting the primer design. [file 12917_2020_2585_MOESM1_ESM.tif]

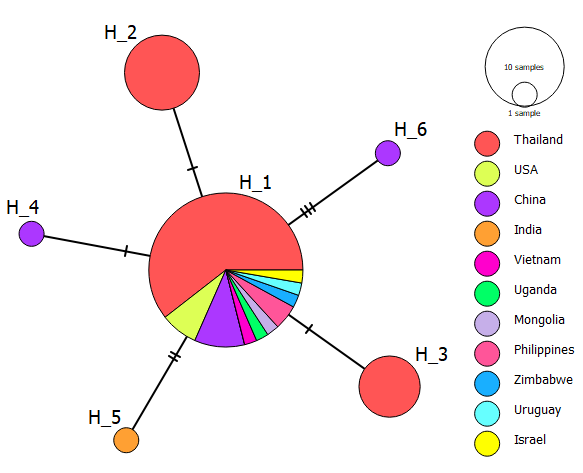

Supplement: Supplementary file 2 — Additional file 2: Figure S2 Median Joining Network of A. marginale based on 16S rRNA haplotype among Thailand and other countries. [file 12917_2020_2585_MOESM2_ESM.tif]
