# Supplementary material for: Molecular detection and genetic characterization of Anaplasma marginale and Anaplasma platys-like (Rickettsiales: Anaplasmataceae) in water buffalo from eight provinces of Thailand
Source: BMC Vet Res. 2020 Oct 8;16:380. doi: 10.1186/s12917-020-02585-z (PMC7542745; doi:10.1186/s12917-020-02585-z)
Supplement: Supplementary file 3 — Additional file 3: Table S1 Oligonucleotide primers used in this study. Table S2 Reference sequences of 16S rRNA and groEL genes from global isolates included in the phylogenetic analyses. [file 12917_2020_2585_MOESM3_ESM.docx]

**Supplementary Tables**

**Table S1.** Oligonucleotide primers used in this study

| Target | Oligonucleotide sequence (5’🡪 3’) | | Amplicon size (bp) | Annealing temp (^o^C) | Reference |
| --- | --- | --- | --- | --- | --- |
|  | Forward | Reverse |  |  |  |
| Anaplasmataceae-*16S rRNA* | cPCR: CAAACTTGAGAGTTTGATCCTGG | ACGATAAGAAAGCCTAAAAGGAGG | 1520 | 55 | This study |
|  | nPCR: TGGCAGACGGGTGAGTAATG | TAAGCCAATTCCCATGGCGT | 1286 | 57 | This study |
| *A. platys groEL* | GAAGAGTATTAAGCCTGAGGAACCGC | GTCGTTGTGTCCTTAGTGATGCGAAC | 825 | 60 | This study |

**Supplementary Table S2** Reference sequences of *16S rRNA* and *groEL* genes from global isolates that were included in the phylogenetic analyses

**(a) *16S rRNA* gene**

| **Species** | **Host (species if any) or source** | **Country of origin** | **Accession No.** |
| --- | --- | --- | --- |
| *Anaplasma marginale* | *Rhipicephalus microplus* | Philippines | JQ839012 |
|  | Cattle | USA | CP001079 |
|  | NA | South Africa | AF414873 |
|  | Cattle | Thailand | KT264188 |
|  | Cattle | China | AJ633048 |
|  | NA | USA | AF311303 |
|  | Buffalo | China | HM538192 |
|  | Cattle | Uganda | KU686794 |
| *A. centrale* | *Rhipicephalus microplus* | Philippines | JQ839010 |
|  | *Rhipicephalus simus* | South Africa | AF414869 |
|  | Cattle | Japan | AF283007 |
|  | Cattle | Italy | EF520690 |
| *A. bovis* | *Haemaphysalis longicornis* | Japan | AB196475 |
|  | Goat | China | JN558819 |
|  | Cattle | China | FJ169957 |
|  | Goat | China | JN558825 |
|  | Goat | China | MH255925-MH255926 |
|  | Cattle | China | MH255927 |
|  | Goat | China | MH255928-MH255933 |
|  | Cattle | China | MH255934 |
|  | Goat | China | MH255935 |
|  | Cattle | China | MH255936-MH255937 |
|  | Cattle | China | MH255941 |
| *A. ovis* | *Dermacentor everestianus* | China: Tibet | JQ917905 |
|  | *Dermacentor niveus* | China: Tibet | JQ917880 |
|  | *Dermacentor niveus* | China: Tibet | JQ917902 |
|  | *Dermacentor niveus* | China: Tibet | JQ917876-JQ917879 |
|  | *Dermacentor niveus* | China: Tibet | JQ917881-JQ917885 |
| *A. phagocytophilum* | *Ixodes ricinus* | Australia | JX173652 |
|  | Goat | China | HQ872464 |
|  | Horse | Sweden | AY527214 |
|  | Deer *(Cervus nippon nippon)* | Japan | AB196721 |
| *A. platys* | Dog *(Canis lupus familiaris)* | Italy | EU439943 |
|  | *Rhipicephalus simus* | China | MH762081 |
|  | Dog | Venezuela | AF399917 |
|  | Dog | Thailand | EF139459 |
| *Rickettsia rickettsii* | NA | France | L36217 |
|  | *Dermacentor andersonii* | USA | U11021 |

**(b) *groEL* gene**

| *Species* | Host (species if any) or source | Country of origin | Accession No. |
| --- | --- | --- | --- |
| *A. marginale* | cattle | USA | CP001079 |
|  | *Rhipicephalus microplus* | Philippines | JQ839003 |
| *A. centrale* | *Rhipicephalus simus* | South Africa | AF414866 |
|  | cattle | Italy | EF520695 |
| *A. ovis* | Goat | Cyprus | FJ460441 |
|  | NA | South Africa | AF441131 |
|  | Sheep | China | CP015994 |
|  | Goat | China | MG869402 |
|  | Sheep | China | KX579069 |
| *A. platys* | Dog *(Canis lupus familiaris)* | Chile | EF201806 |
|  | Dog *(Canis lupus familiaris)* | Argentina | KF826285 |
|  | Dog *(Canis lupus familiaris)* | Uruguay | KX792012 |
|  | Dog *(Canis lupus familiaris)* | Cuba | MK509746 |
|  | Dog *(Canis lupus familiaris)* | Thailand | KU765205 |
|  | Dog *(Canis lupus familiaris)* | Thailand | KU765203 |
|  | *Rhipicephalus sanguineus* | Argentina | KR909453 |
|  | *Rhipicephalus sanguineus* | Thailand | MK660529 |
|  | *Rhipicephalus sanguineus* | Philippines | JN121382 |
|  | *Armigeres subalbatus* | China | KU585944 |
|  | *Rhipicephalus microplus* | China | KX987394 |
|  | *Rhipicephalus sanguineus* | Taiwan | KY581623 |
|  | *Rhipicephalus microplus* | China | MH716435 |
|  | *Anopheles sinensis* | China | KU585930 |
|  | Dog *(Canis lupus familiaris)* | Japan | AY044161 |
|  | Dog *(Canis lupus familiaris)* | Japan | AY077621 |
| *A. phagocytophilum* | Red deer *(Cervus elaphus)* | Spain | HM057225 |
|  | Human | USA | CP000235 |
|  | Hourse | Italy | AY848749 |
|  | Dog *(Canis lupus familiaris)* | Italy | AY848752 |
| *A. bovis* | *Myodes rufocanus* | Russia | JX092099 |
|  | *Haemaphysalis concinna* | Russia | JX092095 |
| *Ehrlichia canis* | *Rhipicephalus evertsi* | South Africa | MG953295 |
|  | Dog *(Canis lupus familiaris)* | China | CP025749 |
|  | Cell culture | USA | U96731 |
|  | *Rhipicephalus sanguineus* | Philippines | JN391407-JN391408 |
